# Nrf2 Signaling in Renal Cell Carcinoma: A Potential Candidate for the Development of Novel Therapeutic Strategies

**DOI:** 10.3390/ijms252413239

**Published:** 2024-12-10

**Authors:** Valentina Schiavoni, Monica Emanuelli, Giulio Milanese, Andrea Benedetto Galosi, Veronica Pompei, Eleonora Salvolini, Roberto Campagna

**Affiliations:** 1 Department of Clinical Sciences, Polytechnic University of Marche, 60020 Ancona, Italy; v.schiavoni@pm.univpm.it (V.S.); m.emanuelli@univpm.it (M.E.); g.milanese@univpm.it (G.M.); a.b.galosi@univpm.it (A.B.G.); v.pompei@staff.univpm.it (V.P.); 2 New York-Marche Structural Biology Center (NY-MaSBiC), Polytechnic University of Marche, 60131 Ancona, Italy

**Keywords:** renal cell carcinoma, RCC, nuclear factor erythroid 2-related factor 2, Nrf2

## Abstract

Renal cell carcinoma (RCC) is the most common type of kidney cancer arising from renal tubular epithelial cells and is characterized by a high aggressive behavior and invasiveness that lead to poor prognosis and high mortality rate. Diagnosis of RCC is generally incidental and occurs when the stage is advanced and the disease is already metastatic. The management of RCC is further complicated by an intrinsic resistance of this malignancy to chemotherapy and radiotherapy, which aggravates the prognosis. For these reasons, there is intense research focused on identifying novel biomarkers which may be useful for a better prognostic assessment, as well as molecular markers which could be utilized for targeted therapy. Nuclear factor erythroid 2-related factor 2 (Nrf2) is a transcriptional factor that has been identified as a key modulator of oxidative stress response, and its overexpression is considered a negative prognostic feature in several types of cancers including RCC, since it is involved in various key cancer-promoting functions such as proliferation, anabolic metabolism and resistance to chemotherapy. Given the key role of Nrf2 in promoting tumor progression, this enzyme could be a promising biomarker for a more accurate prediction of RCC course and it can also represent a valuable therapeutic target. In this review, we provide a comprehensive literature analysis of studies that have explored the role of Nrf2 in RCC, underlining the possible implications for targeted therapy.

## 1. Introduction

Renal cell carcinoma (RCC) is classified as the most common type among kidney cancers, accounting for 90% of new kidney cancer diagnoses with 431,288 new cases diagnosed in 2020 globally [1,2]. The incidence is variable, but the Czech Republic and North America have the highest rate. In particular, in the United States, 63,000 new cases and almost 14,000 deaths per year have been observed [3]. RCC is more often diagnosed in males than in females: the incidence ratio is 2:1 male–female, and male gender is related to more aggressive histological characteristics in terms of stage and grade of tumor [4]. Although RCC may occur at any age, it is diagnosed more frequently in men aged between 60 and 70 years [5]. RCC originates from the proximal renal tubular epithelium and three different subtypes with different histology are described: clear cell (ccRCC), papillary cell (pRCC), and chromophobe renal cell carcinoma (chRCC). Among these, the most common variant is ccRCC, which shows a percentage of incidence of 80% and is accountable for most of the deaths caused by RCC [6]. It derives from epithelial cells of the proximal convolute tubule in the nephron, and its name comes from the observation that these cells are characterized by a clear cytoplasm. Approximately 2–3% of ccRCC cases are hereditary, and in particular, patients with an alteration of the von Hippel–Lindau (*VHL*) gene are most at risk [7]. ccRCC is characterized by a high risk of metastases, particularly in lungs, liver, bones, and lymph nodes [8]. pRCC is the second most common variant and two different subtypes are described: type 1 and type 2, which differ in stage, grade, and prognosis. Type 2, characterized by mutations in fumarate hydratase gene, is highly aggressive compared to type 1, and it has the worst prognosis [9]. chRCC is classified as the third most common subtype of RCC and two variants are reported: classical and eosinophilic. Moreover, it represents the least aggressive RCC subtype due to its low tendency to metastasize. The high mortality rate of RCC, equal to 179,368 deaths worldwide in 2022 (115,600 men and 63,768 women), is related to late diagnosis, which is usually obtained incidentally when the patient undergoes medical tests, such as cross-sectional abdominal imaging, for other reasons [2]. Indeed, more than 50% of patients with a diagnosis of RCC are asymptomatic, and the classical triad (hematuria, flank pain, and palpable abdominal mass) appears when the cancer is at a late stage [10,11]. The tumor, nodes, and metastasis (TNM) staging system is the most used anatomical prognostic factor applied for multiple solid tumors including RCC since 1977 [11]. The letter T refers to the size of the primary tumor, and it is associated with a scale ranging from 1 to 4, defining masses of larger dimensions. N indicates the involvement of regional lymph nodes, and M is associated with the presence of metastases. In particular, stages I and II are limited to the kidney, while stages III and IV indicate tumors that have spread beyond the kidney. The prognosis is associated both with the stage and the histological subtypes: patients with stages I or II show a 5-year survival rate of 80–90%, while an advanced stage correlates with a lower 5-year survival rate [12]. Moreover, the variant with the highest survival rate is pRCC (90%), followed by ccRCC, with a percentage equal to 50–60% that dramatically reduces to 10% when it spreads to other parts of the body, thus being more difficult to treat [11].

RCC incidence increases with age, but risk factors related to lifestyle, such as obesity, hypertension, alcohol consumption, and smoking, contribute to up to 50% of tumor development [13]. Occupational exposure to trichloroethylene (TCE), a degreasing agent considered highly carcinogenic, has been associated with the development of the ccRCC subtype [14,15]. Moreover, hereditary diseases must be taken into account as risk factors, such as VHL syndrome, which represents the most common inherited disease leading to ccRCC development through the activation of vascular endothelial growth factor (VEGF). Since about 60% of sporadic ccRCCs share the same pathogenesis, new therapies inhibiting VEGF have been developed to treat both heritable and sporadic cases of ccRCC [16]. Over the past three decades, the RCC treatment landscape has evolved significantly. Chemotherapy and radiotherapy are not effective due to the intrinsic resistance of all subtypes. Lack of sensitivity to chemotherapy has stimulated research on new treatment options. Some studies have demonstrated that stereotactic ablative body radiation (SAbR) is an emerging non-invasive alternative treatment for primary RCC tumors. Generally, surgery represents the gold standard for the treatment of localized RCC, in particular radical or partial nephrectomy. The latter has now been recognized as the surgical standard for management of T1 (≤7 cm) renal masses and for T2 (>7 cm and limited to kidney) lesions in selected patients, with the aim of preserving kidney function. When the tumor is at an advanced stage (stage III–IV), immunotherapy, in particular immune checkpoint inhibitor therapy, and targeted therapy with antiangiogenic agents (monoclonal antibodies, kinase inhibitors, VEGF, and mTOR inhibitors) are useful treatment options [15,17].

RCC is characterized not only by late diagnosis and high incidence but also by a remarkable tendency to metastasize and an intrinsic resistance to chemotherapeutic drugs; the identification of novel biomarkers for early diagnosis, a more accurate prognosis, and the development of new targeted therapies to manage the disease is therefore a crucial goal.

Nuclear factor erythroid 2-related factor 2 (Nrf2) is a transcriptional factor that regulates the gene expression of a wide variety of antioxidant cytoprotective enzymes [18,19,20]. For cancer cells, it is important to maintain tolerable levels of reactive oxygen species (ROS) through multiple signaling pathways in order to preserve their malignancy [21,22]. Indeed, one strategy to fight cancer cells is to use anticancer drugs that induce ROS overproduction [23,24,25,26,27,28]. Several studies reported that different types of cancers are characterized by chronic activation of Nrf2, demonstrating a transformation from a cytoprotective pattern to a pro-tumorigenic one [29]. Moreover, Nrf2 can activate oncogenes unrelated to ROS detoxification. In cancer cells, Nrf2 activation therefore has a dual function: neutralization of ROS production and, at the same time, an increase in cancer cell survival and resistance to treatment through the activation of treatment-resistance proteins and oncogenes [30,31]. In particular, the transitory activation of Nrf2 in kidney tissue can lead to kidney injury caused by an accumulation of ROS, which are known to promote tumorigenesis and tumor progression. Moreover, chronic activation of Nrf2 in RCC cells results in an increased metastatic and proliferative potential. There are not yet specific inhibitors of Nrf2 due to the similar structure of this molecule compared with other members of bZip family [32]. Thus, research should focus both on designing high specific inhibitors for Nrf2 and on identifying different pathways which are implicated in the aberrant activation of Nrf2 in order to indirectly suppress the Nrf2 activation pathway. Indeed, compared to other malignancies, RCC is characterized by limited targeted therapeutic options. In this review, we will analyse the role of the Nrf2 signaling pathway, focusing on RCC, and we will evaluate its potential use as a diagnostic/prognostic biomarker and a therapeutic target.

## 2. Nuclear Factor Erythroid 2-Related Factor 2 (Nrf2)

**Figure 1:**
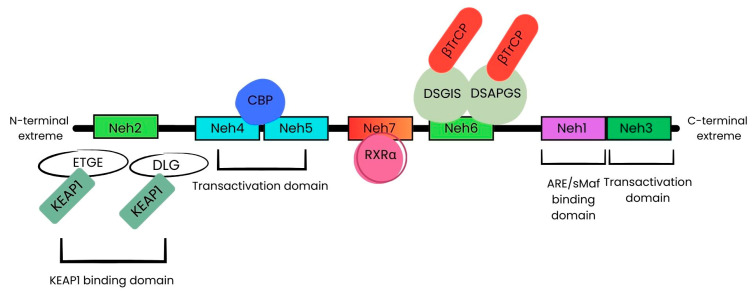
Schematic representation of *Nrf2* domains structure. Neh: NRF2-ECH homology domains; KEAP1: Kelch-like-ECH-associated protein 1; ARE: antioxidant response elements; CBP: CREB-binding protein; βTrCP: β-transducin repeat-containing protein; RXRα: retinoic X receptor.

Nrf2 is a transcriptional factor encoded by the gene nuclear factor erythroid 2 like 2 (*NFE2L2*). It belongs to the Cap’n’Collar (CNC) subfamily of basic leucine zipper (bZip) transcription factors, also including Nrf1 and Nrf3. *Nrf2* is characterized by the presence of seven NRF2-ECH homology (Neh) domains showing different functions. In particular, bZip in the Neh1 domain heterodimerizes with small musculoaponeurotic fibrosarcoma proteins (sMAF) K, G, and F, and this heterodimerization allows it to interact with antioxidant response elements (ARE) for the activation of gene transcription. Neh2, instead, contains two motifs, ETGE and DLG, which bind to the Kelch domain of Kelch-like-ECH-associated protein 1 (KEAP1), thus leading to the ubiquitination and degradation of Nrf2. The Neh3 domain works as a transcriptional activation domain, as well as Neh4 and Neh5, which interact with CREB-binding protein (CBP). The Neh6 domain contains DSGIS and DSAPGS, two redox-independent degrons, that bind to E3 ubiquitin ligase β-transducin repeat-containing protein (βTrCP), responsible for Nrf2 degradation. Lastly, the Neh7 domain, interacting with retinoic X receptor alpha (RXRα), inhibits Nrf2 cytoprotective activity (Figure 1) [33,34].

**Figure 2:**
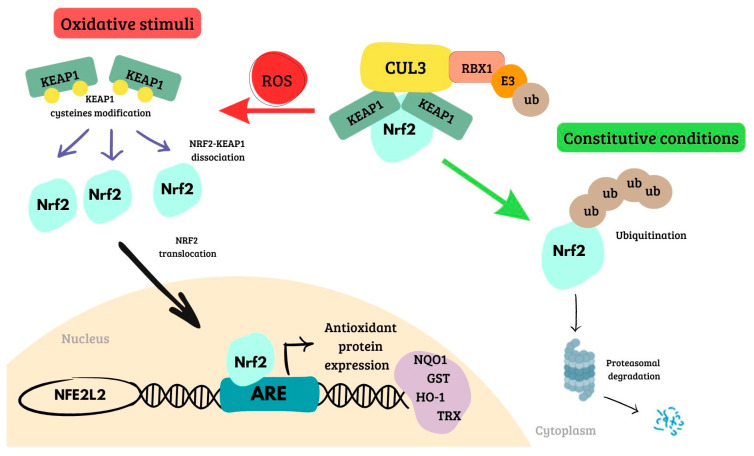
KEAP1–Nrf2-ARE pathway in constitutive condition and under oxidative stimuli. Nrf2 is continuously produced: in constitutive conditions, it is retained in the cytoplasm by KEAP1, which promotes its degradation via the CUL3-RBX1 ubiquitin ligase complex; under oxidative stimuli, ROS modify KEAP1 cysteines, leading to Nrf2 translocation to the nucleus where it activates the expression of genes involved in the oxidant response. KEAP1: Kelch-like ECH-associated protein 1; Nrf2: nuclear factor-erythroid 2; ARE, antioxidant response elements; CUL3: cullin 3; RBX1: RING-box protein 1; NFE2L2: *Nrf2* gene; Ub: ubiquitin; NQO1: NAD(P)H quinone oxidoreductase 1; GST: glutathione S-transferases; HO-1: heme oxygenase-1; TRX: thioredoxin; ROS: reactive oxygen species.

Cells counteract harmful effects of ROS through the activation of *Nrf2*. In the basal state, Nrf2 levels are low due to its bond with KEAP1, which interacts with the ETGE and DLG motifs in the Neh2 domain of *Nrf2*. CULLIN 3 (CUL3) and RING-box protein 1 (RBX1)/E3-ubiquitin ligase are bound to KEAP1 to form the KEAP1/CUL3/RBX1 E3-ubiquitin ligase complex, which is responsible for the proteasomal degradation of Nrf2 [35]. Generally, in response to oxidant stimuli, electrophiles and ROS react with sensor cysteines of KEAP1 and inhibit the constitutive degradation of Nrf2 [36]. It is therefore stabilized and translocates to the nucleus, allowing the transcription of its downstream targets binding to ARE regions in the promoter region of antioxidant genes (Figure 2) [37]. Moreover, the Nrf2 signaling pathway is influenced by the phosphatidylinositol 3-kinase (PI3K)/AKT pathway. This regulation involves phosphorylation by glycogen synthase kinase-3 beta (GSK-3β), leading to the degradation of Nrf2 via a β-transducin repeats-containing protein (β-TrCP)-CUL1 mechanism [38]. Instead, when GSK-3β is inhibited by the AKT-mediated phosphorylation, Nrf2 is not phosphorylated and the β-TrCP-CUL1 E3 ubiquitin ligase complex does not recognize Nrf2, thus preventing its degradation [39].

Genes regulated by Nrf2 encode enzymes involved in the detoxification of phase I, II, and III drugs and in the metabolic elimination of pro-oxidants. Phase I enzymes, which include carbonyl reductases (CBRs), aldo-keto reductases (AKRs), NAD(P)H quinone oxidoreductase 1 (NQO1), aldehyde dehydrogenase 1 (ALDH1), and various cytochrome P450s (CYPs), such as CYP2a5 and CYP2b6, mediate the oxidation, hydrolysis, and reduction of different xenobiotics [40,41]. Phase II enzymes can modify metabolites generated by phase I enzymes by attaching to them hydrophilic molecules in order to increase their solubility and promote their excretion. Among these enzymes are heme oxygenase-1 (HO-1), UDP-glucuronosyltransferases (UGTs), and glutathione S-transferases (GSTs) [42,43]. Phase III enzymes include multidrug resistance-associated proteins (MDR), breast cancer-resistant protein (BCRP), and adenosine triphosphate (ATP)-binding cassette g5 (ABCG5) and g8 (ABCG8) which are part of the drugs efflux transporters [44]. Specifically, Nrf2 primarily targets genes that are involved in the biogenesis of reducing agents and the regeneration of their oxidized forms, including glutathione (GSH) and thioredoxin (TRX), as well as stress-responsive proteins and those that play a role in the breakdown of superoxides and peroxides [45]. Recently, different studies have described new Nrf2 target genes and have discovered new functions in addition to its redox-regulation capacity. Moreover, Nrf2 is now considered a promising target in the field of cancer metabolism, prevention, and treatment.

## 3. Nrf2 and Cancer

In cancer cells, oxidative stress plays an important role in tumor development through the impairment of pathways involved in DNA repair and cell survival [46]. Superoxide (O2^●−^), hydroxyl radical (OH^−^), and hydrogen peroxide (H_2_O_2_) are the main ROS which can damage DNA, lipids, and proteins. Antioxidant enzymes, such as catalases, superoxide dismutase, thioredoxins, peroxiredoxins, reductases, peroxidases, and paraoxonases, protect the cell from ROS-induced damage [47]. Moreover, cells can counteract ROS production through non-enzymatic molecules, including glutathione, coenzyme-Q, and lipoic acid. Inflammation plays a key role in the onset and progression of several diseases, including cancer [48,49]. Tumors are complex structures composed of heterogeneous cell populations that evolve in response to genetic and epigenetic changes, as well as metabolic shifts in their microenvironment [50,51]. This dynamic nature of cancer allows tumor cells to adapt and frequently develop resistance to treatment. Moreover, individual differences among patients and heterogeneity within tumors often lead to different responses to therapies. A promising anti-cancer strategy involves the simultaneous inhibition of antioxidant circuits and metabolic pathways that maintain the redox balance of malignant cells [52,53].

It is well established that metabolic changes in cancer cells, which drive their proliferation and growth, also lead to increased ROS generation. The raise in ROS production is counterbalanced by antioxidant mechanisms that prevent cell death. Since many conventional anti-cancer therapies rely on ROS accumulation for cytotoxicity, the intensified antioxidant defense of cancer cells is a major player in treatment resistance. On the other hand, interfering with the redox balance of cancer cells can induce oxidative stress-dependent cell death [54].

The Nrf2 signaling pathway, which plays a critical role in tumorigenesis, malignant progression, and drug sensitivity, has emerged as a key therapeutic target. Consequently, Nrf2 has driven attention both for its potential activation to prevent ROS-driven carcinogenesis and for the development of Nrf2 inhibitors to overcome resistance to treatment [51].

Recently, Nrf2 has been identified as a key modulator of oxidative stress response, and it has been shown to play a dual role in tumor progression, since it suppresses cell damage caused by oxidative stress and exerts an anti-inflammatory function, thus inhibiting tumor initiation, as demonstrated in studies showing that *Nfe2l2*^−^/^−^ mice are more susceptible to cancer induced by chemical and radiation exposure, an effect that is likely to be related to an impaired oxidative stress response [34,55]. On the other side, Nrf2 allows cancer cells to modify their metabolism in order to enhance metabolic reprogramming and support rapid cell proliferation and invasiveness. Moreover, *Nrf2* activation is considered a target mechanism for overcoming therapy resistance, since different anticancer drugs exert their cytotoxic effect by inducing ROS overproduction in cancer cells. More than 600 somatic mutations in the *NFE2L2* gene have been identified across various cancers, with the majority affecting the DLG and ETGE motifs, abolishing the interaction of Nrf2 with KEAP1 [56,57]. These mutations lead to *Nrf2* stabilization and persistent activation, which is often associated with a poor prognosis. Activating *Nrf2* mutations have been described in lung cancer, liver cancer, ovarian cancer, head and neck cancer, kidney cancer, breast cancer, and esophageal cancer, but loss-of-function mutations in *KEAP1* and *CUL3* also occur frequently [58,59,60,61,62].

Generally, *Nrf2* activation is a negative prognostic factor in cancer; indeed, it is involved in several key cancer-supportive functions, including cancer cells proliferation through the upregulation of anabolic metabolism and by acting on epidermal growth factor receptor signaling. Moreover, it confers resistance to chemotherapy through the induction of drug detoxification enzymes and efflux transporters expression, and it induces the expression of ROS-scavenging enzymes and antiapoptotic B-cell 2 (Bcl-2) family members (Bcl-2 and Bcl-xL), thus conferring resistance to apoptosis [63,64]. In addition, Nrf2 confers resistance to ferroptosis by inducing key enzymes of glutathione synthesis and metabolism, suppresses p53-induced senescence, and protects telomeres from DNA damage caused by oxidative stress [65,66]; furthermore, it induces angiogenesis and activates invasion and metastasis through the downregulation of E-cadherin expression and by promoting epithelial-to-mesenchymal transition (EMT) [67]; finally, it allows cancer cells to avoid immune destruction and it inhibits the production of antitumor cytokines, such as IFN-γ [68]. The impact of Nrf2 activation on tumor immunity has recently gained significant attention, particularly in relation to both cancer intrinsic mechanisms and immune cell-related activation. Since immune therapies, such as immune checkpoint inhibitors, have become standard treatments for many cancers, including RCC, understanding the factors that influence their efficacy is crucial. Notably, the relationship between *KEAP1* mutations and a low number of tumor-infiltrating lymphocytes suggests a link between Nrf2 hyperactivity and immune evasion. For example, in squamous cancers, Nrf2-hyperactive tumors exhibit a molecular phenotype characterized by *SOX2* amplification and *CDKN2A/B* loss, both associated with a reduced presence of lymphocytes and macrophages, regardless of the specific mechanism of Nrf2 activation [38].

Moreover, Nrf2 has been shown to regulate the immune checkpoint molecule PD-L1 in melanoma, and inhibition of both Nrf2 and PD-1 together leads to a synergic inhibition of tumor growth. Further research has demonstrated that the loss of KEAP1 results in a reduction in tumoral CD8+ T cells and an increase in M2 macrophages. Nrf2 also inhibits the type I interferon response directly in vitro via suppression of the stimulator of interferon genes. Additionally, it has been suggested that Nrf2-associated immune evasion may involve inhibition of proinflammatory cytokines, enhanced antioxidant capacity, and the production of immunomodulatory metabolites [38].

Given its key role in carcinogenesis, the Nrf2 pathway is considered a target to boost chemotherapy in different cancer types [65]. Both activators and inhibitors of Nrf2 have been considered in cancer prevention and therapy, respectively [69]. A main challenge is the possibility of selectively activating Nrf2 in healthy tissues while inhibiting it exclusively in cancer cells. Most Nrf2 activators are redox-active or electrophilic compounds that react with cysteine residues of KEAP1 but are not specific to it. Studies in animal models have demonstrated that these activators enhance detoxification of carcinogens and exert a protective effect on tissues, particularly on the liver [70]. Examples of Nrf2 activators are sulforaphane (from cruciferous vegetables), which protects against lung and pancreatic cancers [71]; oltipraz, a synthetic compound that inhibits cancer formation in rodent models and may mitigate non-alcoholic steatohepatitis [72,73]; and dimethyl fumarate (DMF), FDA-approved for multiple sclerosis, with potential tumor-preventive effects [74]. Electrophilic activators can lack specificity as they can react with nucleophilic cysteine residues of other proteins, including glyceraldehyde 3-phosphate dehydrogenase (GAPDH) [75]. Non-electrophilic compounds are instead more selective and disrupt protein–protein interactions (PPIs) (Nrf2:KEAP1 or KEAP1:CUL3) [70,76]. Pharmacological inhibition of Nrf2 is also a promising approach for cancer therapy. Although some Nrf2 inhibitors have been developed, none of them have given satisfactory results. Due to the low specificity of both possible treatments, future studies should focus on developing more specific agents targeting Nrf2 [34].

## 4. The Nrf2 Signaling Pathway in RCC

Pathological conditions (hypertension and hyperglycemia) or exogenous toxic stimuli (chemotherapy or nephrotoxins) can induce kidney injury through the accumulation of ROS, leading to oxidative stress, which represents the cause of the initiation and progression of acute and chronic kidney disease. In basal conditions, Nrf2 is negatively regulated by KEAP1, which keeps it at low levels. When pathological conditions or exogenous toxic stimuli arise, there is a transitory activation of Nrf2 [77]. Generally, the accumulation of oxidative damage results in malignant transformation and then in tumor development [78]. Nrf2 induction before the development of cancer is, on the contrary, an important chemopreventive strategy. However, in RCC, the constitutive activation of Nrf2, resulting from modifications of the KEAP1–Nrf2-CUL3 complex, enhances cancer cell proliferation, survival, metastatic potential, and resistance to therapies [79]. *Nrf2*’s dual role in normal and malignant cells can be explained through the different transcription signatures activated by Nrf2 or through ARE-domains, since cancer-ARE are localized in more accessible chromatin regions compared to noncancer-ARE. Premalignant cells are highly influenced by inflammatory and stromal cells within their microenvironment and have not yet accumulated enough DNA damage to become autonomous. Consequently, enhancing Nrf2 activity, which helps to mitigate inflammatory responses and oxidative stress, may be beneficial in premalignant stages, contributing to suppressing carcinogenesis. The timing of Nrf2 activation is crucial: while Nrf2 activity is beneficial in the early phases of tumorigenesis, when the host organism is attempting to suppress premalignant changes, it becomes undesirable in later stages. Indeed, in advanced tumors, Nrf2 activation may promote resistance to treatment in malignant cancer cells [80].

The chronic Nrf2 activation in cancer enhances chemoresistance. For instance, cisplatin, which induces DNA adducts’ formation and oxidative stress, causes the activation of the Nrf2 pathway with a reduction of drug cytotoxicity in kidney epithelial cells, thus suggesting a possible combinatory treatment to prevent chemotherapy side effects. ccRCC and pRCC contrast radio- and chemotherapy-induced cytotoxicity through the chronic hyperactivation of Nrf2 and the expression of antioxidant genes and drug-metabolizing enzymes [81,82].

Increased oxidative stress and activation of Nrf2/ARE pathway characterize aggressive tumors. Nrf2 pathway results are altered in 4.7% of all RCC subtypes [83]. Mutations that involve the Nrf2 coding gene *NFE2L2*, as well as genes encoding regulatory proteins, such as KEAP1 and CUL3, and those that encode the transcriptional targets of Nrf2, are mutually exclusive [84].

**Figure 3:**
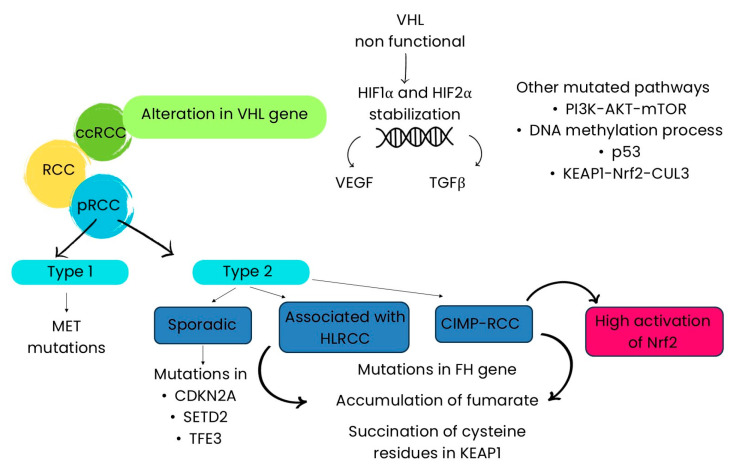
Nrf2 signaling pathway in RCC. RCC: renal cell carcinoma; ccRCC: clear cell renal cell carcinoma; pRCC: papillary renal cell carcinoma; HLRCC: hereditary leiomyomatosis and renal cell carcinoma; CIMP-RCC: CpG Island Methylator Phenotype-RCC; *VHL*: von Hippel–Lindau; HIF: hypoxia-inducible factor; VEGF: vascular endothelial growth factor; TGFβ: transforming growth factor; *CDKN2A*: cyclin-dependent kinase inhibitor 2A; *SETD2*: histone lysine methyltransferase; *TFE3*: transcription factor E3; *FH*: fumarate hydratase.

ccRCC is hereditary in 2–3% of cases, and 90% of patients with sporadic ccRCC show a genomic alteration in the short arm of chromosome 3, where the gene *VHL* is located. In normoxic conditions, VHL is involved in the proteasomal degradation and polyubiquitination of hypoxia-inducible factor (HIF). The degradation of VHL results in the stabilization of HIF1α and HIF2α in normal oxygen conditions, leading cells to a state of pseudohypoxia. This involves the activation of HIF-dependent transcription genes (glycolytic enzymes, VEGF and TGF-β) which are included in the pro-tumorigenic adaptation [85,86]. Other newly identified pathways and components frequently mutated in ccRCC include the PI3K-AKT-mTOR signaling pathway, DNA methylation processes, p53-related pathways, mRNA processing mechanisms, and the KEAP1-NRF2-CUL3 complex [87]. While genetic alterations in the KEAP1–Nrf2 pathway have been reported in only a small number of ccRCC patients, a notable association has been found between *KEAP1* promoter methylation and the histological characteristics of ccRCC. These genetic and epigenetic modifications contribute to the upregulation and activation of the *Nrf2* gene and its protein product [88]. Concerning pRCC, it can be distinguished in type 1 and type 2. Type 1 pRCC shows mutations that activate oncogenes, in particular somatic or germline mutations in the *MET* gene, which implies higher cell proliferation, survival, and migration. Type 2 can occur either sporadically or in association with hereditary leiomyomatosis and renal cell carcinoma (HLRCC). The sporadic one shows mutations in cyclin-dependent kinase inhibitor 2A (*CDKN2A*), histone lysine methyltransferase (*SETD2*), and transcription factor E3 (*TFE3*), while the hereditary one shows mutations in the germ line fumarate hydratase (*FH*) gene. CpG Island Methylator Phenotype-RCC (CIMP-RCC) has been recently identified as a new subgroup of type 2 pRCC, characterized by mutations in the *FH* gene. Moreover, it shows a different metabolic profile among pRCC subtypes, resulting in a strong decrease in Krebs cycle gene expression and an increase in the signature of ribose metabolism [16]. Nrf2 is overexpressed in all pRCC subtypes, showing high activation in CIMP-RCC, medium in type 2 pRCC, and low in type 1. However, mutations in genes encoding Nrf2 as well as in its negative regulators fail to completely explain the overexpression of the Nrf2 transcriptional signature in different tumor types. Indeed, post-transcriptional mutations, which target Nrf2 directly or its interacting proteins, can modulate Nrf2 activity. For example, fumarate is able to succinate KEAP1 and DJ-1 in type 2 CIMP-RCC and HLRCC, which are FH-deficient tumors (Figure 3) [89,90].

Fumarate highlights the context-dependent role of Nrf2 in carcinogenesis. At physiological levels, fumarate, as a key metabolite in the Krebs cycle, is fundamental for life. As well as other Nrf2 activators, when administered at appropriate pharmacological doses, fumarate can prevent the development of cancer in various organs in animal models. However, when intracellular fumarate levels become chronically elevated due to mutations in *FH*, it acts as a carcinogen. This mirrors the effects of Nrf2 hyperactivation observed in several tumors, including RCC [80].

## 5. Nrf2 Modulators in RCC

### 5.1. Nrf2 Hyperactivation Supports Epithelial-to-Mesenchymal Transition

Nrf2 is implicated in EMT, a dynamic and reversible mechanism in which cells acquire the ability to migrate and a malignant potential that are fundamental in the metastasis mechanism [91,92,93,94]. Nrf2 activation in cancer tissues supports both drug resistance and the EMT process because it compromises E-cadherin expression, resulting in a reduction of N-cadherin and metalloprotease production [66]. Mutations in the *NFE2L2* gene, found in some tumors, show constitutive activation of Nrf2 leading to increased proliferation, anchorage-independent growth, and metastatic potential, dependent on mTORC1 activation. It has been demonstrated that expressing the mutant *Nrf2* gene in human embryonic kidney (HEK)-293 cells gives them oncogenic and metastatic properties. Since Nrf2 maintains cancer cells in a hybrid state, preventing complete transition (epithelial-to-mesenchymal hybrid transition), as occurs in RCC, cancer cells display higher metastatic potential and develop drug resistance [95,96]. It has been reported in transformed cells of squamous cell carcinoma that transforming growth factor (TGF) β1 induces Nrf2 stabilization in a p-21 dependent manner, resulting in radio- and chemo-resistance through the regulation of glutathione metabolism [97,98]. RCCs characterized by Nrf2 hyperactivation might show the same Nrf2-driven mechanism. Nonetheless, in healthy kidney tissue, Nrf2 opposes the EMT process, which is usually activated during organogenesis and development, but also in response to organ damage [99]. Instead, in renal fibrosis, a common feature of diabetic nephropathy, TGFβ1 activation and ROS accumulation support EMT-dependent fibrosis, which results in loss of function and onset of chronic kidney disease (CKD). Transient activation of Nrf2 blocks this mechanism, and in fact, Nrf2 activators, such as sulforaphane, epigallocatechin-3-gallate, and curcumin, may be a good therapeutic option for acute and CKD [100,101,102,103] (Table 1).

### 5.2. Post-Translational Modifications of the KEAP1–Nrf2 Pathway

Post-translational modifications of KEAP1 have an important role in renal cancer progression (Table 1) [107]. In particular, HLRCC is characterized by mutations in *FH* that lead to an accumulation of fumarate, resulting in the succination of cysteine residues in KEAP1 [108]. This prevents Nrf2 ubiquitination, evolving in uncontrolled upregulation of Nrf2 target genes [89]. Moreover, constitutive activation of Nrf2 in HLRCC is caused by loss of function mutation in *CUL3* and gain of function mutations in Nrf2 [61]. Besides post-translational modifications involving KEAP1, Nrf2 also undergoes direct post-translational modifications that affect its subcellular localization and stability. Specifically, as also previously reported, Nrf2 can be regulated by alternative mechanisms, such as phosphorylation performed by various protein kinases, including protein kinase C (PKC), PI3K/Akt, GSK-3β, and JNK. The PI3K/AKT pathway is central to RCC, exhibiting high mutation rates [35]. Notably, mutations in the *PI3K/AKT* and *Nrf2* pathways often co-occur across various tumors [109]. Activation of the PI3K pathway has been linked to Nrf2 accumulation and metabolic changes that enhance cell proliferation and oxidative stress protection. In both transformed renal adenocarcinoma cells and normal renal tubular epithelial cells, insulin activates the PI3K/AKT pathway, leading to Nrf2 phosphorylation, nuclear translocation, and production of the antioxidant enzyme HO-1. This PI3K/AKT-mediated induction of Nrf2 operates independently of PKC and of extracellular signal-regulated kinase (ERK) and p38-MAPK [104]. Furthermore, PI3K/AKT promotes Nrf2 nuclear localization and stability by inhibiting GSK-3β. GSK-3β regulates Nrf2 degradation and nuclear export through phosphorylation mechanisms. Its modulation is crucial for normal kidney tissue response to stress; hyperactivation of GSK-3β in conditions like acute kidney injury impairs Nrf2 accumulation, reducing antioxidant gene induction and contributing to oxidative damage and chronic kidney disease [32].

Although transient Nrf2 activation can be beneficial for normal tissues, it was demonstrated that chronic hyperactivation may promote malignancy. For instance, salvianolic acid treatment activates AKT, inhibiting GSK-3β, which leads to Nrf2 nuclear accumulation and expression of protective genes against oxidative stress in chronic kidney disease [32]. In addition to kinases that directly modify components of the KEAP1–Nrf2 axis, there are also factors that can destabilize their interaction, resulting in alternative activation of downstream signaling pathways. ccRCC is characterized by copy number gains on chromosome 5q, leading to *SQSTM1* oncogene overexpression [110]. *SQSTM1* encodes p62, a multifunctional protein that acts as an adaptor to promote the autophagic degradation of specific proteins [106]. It is known that p62 interacts with signaling molecules, enhancing the activity of downstream effectors, including Nrf2, NF-κB, and mTOR. p62 binds to KEAP1, preventing the degradation of Nrf2. Acute loss of VHL in ccRCC can induce senescence due to oxidative stress, a process that p62 may help to mitigate, contributing to the resistance of ccRCC cells to conventional therapies. *SQSTM1* is a Nrf2 target gene, potentially establishing a positive feedback loop where p62 sequesters KEAP1 and triggers its autophagic degradation, thereby sustaining Nrf2 activation. This mechanism relies on the direct interaction between the KIR domain of p62 and the DC domain of KEAP1. When p62 is overexpressed or phosphorylated at Ser349, it can bind the DC domain of KEAP1 and the DLG domain of Nrf2, thus facilitating Nrf2 release for nuclear translocation. This post-translational modification is dependent on kinases that are normally activated by stress stimuli in healthy tissues, but in cancer, these phosphorylating enzymes are often mutated or hyperactivated [106,111].

### 5.3. Mutations in the NFE2L2 Gene and Epigenetic Modulators

Mutations in the *NFE2L2* gene occur in various tumor types, leading to the activation of Nrf2 targets. Additionally, certain residues of the protein that interact with KEAP1 are modified. These mutations activate the CNC—basic leucine zipper protein (bZIP) transcription factor and have been reported across different cancers, resulting in increased Nrf2-ARE transcription [112]. Furthermore, single nucleotide polymorphisms (SNPs) in the *NFE2L2* promoter can significantly repress *Nrf2* transcription and activity. In particular, the SNP rs6721961 seems to affect Nrf2 expression. Activation of the Nrf2-ARE pathway is associated with an aggressive form of pRCC type 2. To investigate the impact of Nrf2 signaling in human ccRCC, *Nrf2* gene mutations, the rs6721961 SNP, and Nrf2 protein expression were examined in patients with metastatic ccRCC. Additionally, the study investigated how these factors correlate with responses to adjuvant VEGF-targeting therapy and overall survival. Yamaguchi et al. studied the association between ccRCC and SNP rs6721961 in the promoter of *NFE2L2* gene and demonstrated that both homo- and heterozygosity cause increased expression of the protein associated with a worse response to VEGF-targeted therapy and a shorter overall survival [113].

The KEAP1–Nrf2 signaling pathway is subject not only to transcriptional, translational, and post-translational regulation but also to an epigenetic regulation. Epigenetic modifications are hereditable alterations in gene expression which do not involve changes in the primary DNA sequence and that are implicated in the oxidative stress response [114]. In particular, epigenetic modulation of *KEAP1* in ccRCC is the primary mechanism, which accounts for 48.6% of cases. This highlights the KEAP1–NRF2 axis as a key driver in renal cancer [115]. The study of Fabrizio et al. investigated the role of *KEAP1* hypermethylation in RCC, focusing on its epigenetic silencing mechanisms. Analysis of 89 surgical RCC samples revealed that aberrant *KEAP1* promoter methylation occurred in 49% of ccRCC cases [87]. The findings of this study were also particularly important due to the adequate number of analyzed samples. This finding was supported by TCGA data from two cohorts (481 ccRCCs and 265 papillary RCCs). The study demonstrated a significant inverse Pearson correlation between *KEAP1* methylation and transcription levels, with pharmacological treatment (5-azacytidine) restoring KEAP1 expression in three of the four ccRCC cell lines used in the study (ccRCC FG-2, ccRCC FW, and ccRCC 5). In patients with solid tumors, nuclear accumulation of Nrf2 and low KEAP1 expression are linked to poor outcomes, though this has been underexplored in renal cancer [116,117]. A study based on 37 ccRCC cases with follow-up data available showed no significant correlation between epigenetic abnormalities and disease progression; however, the small sample size could be a bias for this study due to the low statistical power [87]. To strengthen the analysis, they examined 450 K methylation array data for *KEAP1* in an independent cohort of 481 ccRCC patients, finding a strong association between *KEAP1* promoter hypermethylation and higher tumor grading and staging. This suggests that *KEAP1* hypermethylation could be a predictor of patient survival. Findings indicate that *KEAP1* epigenetic modulation via CpGs promoter hypermethylation is a key mechanism driving deregulation of KEAP1 in ccRCC. This supports the hypothesis that the KEAP1–Nrf2 pathway plays a significant role in ccRCC subtypes with specific epigenetic changes, suggesting that Nrf2 might be a promising pharmacological target [88].

There is increasing evidence that natural molecules, such as phytochemicals in vegetables and medicinal herbs, exert anti-carcinogenic effects through epigenetic regulation of *Nrf2*. In particular, it has been confirmed that miR-200a-3p/141-3p directly binds to the 3′-UTR of *KEAP1*, leading to a significant dysregulation of the Nrf2/KEAP1 pathway in both renal tumorigenesis and ovarian cancer cells. This dysregulation can be effectively inhibited by phenethyl-isothiocyanate (PEITC), resulting in a reduction in the oxidative stress response [118].

The inactivation of FH, resulting in the accumulation of intracellular fumarate, causes the activation of Nrf2 in hereditary pRCC type 2 [89]. Fumarate covalently modifies KEAP1, making it unable to bind Nrf2. It has been shown that there is a convergence of somatic mutations in sporadic pRCC type 2 with the *FH* mutation in hereditary pRCC type 2. Although the mechanism of Nrf2 activation in sporadic pRCC type 2 has yet to be determined, Nrf2 activation could be the consequence of a somatic mutation in genes that directly regulate *Nrf2* transcription activity, including *CUL3* and *SIRT1* [61]. Fumarate, therefore, behaves as an oncometabolite, and its accumulation is responsible for the expression of EMT-related transcription factors in renal epithelial cells, inhibiting the antimetastatic miRNA miR200ba429 [89]. The epigenetic and phenotypic changes observed can be replicated by incubating FH-proficient cells with cell-permeable fumarate. In RCC patients, the loss of FH is linked to reduced levels of miR-200 and an EMT signature, which correlates with worse clinical outcomes. These findings suggest that the loss of FH and the resulting accumulation of fumarate play a role in the aggressive characteristics of FH-deficient tumors [89].

## 6. Therapeutic Strategies

DMF is a drug used to treat relapsing forms of multiple sclerosis and psoriasis, and is being tested in clinical trials for the treatment of several types of cancer, since it is linked with a wide range of pathways and kinases involved in tumor progression, such as the KEAP1–Nrf2, NF-κB, ERK, and MAPK pathways [119]. Nrf2 signaling activation can be adjusted by modulating DMF concentration. In fact, it has been shown that low doses of DMF can exert cytoprotective effects, with consequent tumor progression, caused by the activation of the Nrf2 antioxidant pathway and impairment of KEAP1 binding. However, high doses of DMF also result in the succination of DJ-1, as well as KEAP1. DJ-1 is a protein encoded by the *PARK7* gene which has antioxidant properties and is capable of binding Nrf2, regulating Nrf2-dependent antioxidant signaling through the prevention of its association with KEAP1. Hence, DMF is not only an activator but also an inhibitor of Nrf2 and DJ-1, and it could be useful for the design of a new therapeutic strategy for the treatment of different types of cancers, including RCC [90].

Special AT-rich binding protein-2 (SATB2), a nuclear matrix-associated protein (NMP), has been shown to be aberrantly expressed in RCC [120,121]. It was demonstrated that SATB2 could induce chromatin rearrangement in specific DNA sequences through the recruitment of chromatin-remodeling proteins to activate or suppress gene transcription. SATB2 in RCC is associated with poor survival; its increase promotes tumorigenesis through mechanisms involving yes association protein (YAP) and Nrf2, enhancing antioxidant interaction and chromatin remodeling [120]. Contrary to what was expected by Jin et al., SATB2 did not affect Nrf2 levels but regulated its transcriptional activity on target genes, occupying more than 80% of Nrf2 binding sites in the genome. SATB2 also recruited the SWItch/Sucrose Non-Fermentable (SWI/SNF) complex to facilitate DNA accessibility, thereby influencing oncogenesis. *SATB2* deletion increased ROS-induced apoptosis and reduced tumor proliferation. Inhibition of SATB2 has been shown to lower tumor proliferation and increase sensitivity to chemotherapy. The results suggest that targeting SATB2 and associated SWI/SNF complexes may represent a promising therapeutic strategy for the treatment of RCC with high YAP levels, although the study was conducted only in cell models and thus should be further validated in in vivo settings [120].

Bone morphogenetic proteins (BMPs) are known to be involved in kidney cancer proliferation and drug tolerance. Yu et al. investigated the role of BMP8A in ccRCC. BMP8A is highly expressed in ccRCC, and thus the authors hypothesized that it could enhance Nrf2 activation, leading to increased tripartite motif-containing protein 24 (*TRIM24*) transcriptional activity. This mechanism may contribute to ccRCC progression and resistance to therapies. In particular, TRIM24 is implicated in several cancers due to its role in modulating transcriptional pathways [122]. TRIM24 acts as a coactivator for various nuclear receptors and is influenced by histone modifications, affecting tumorigenesis-related gene expression. This study demonstrated that *TRIM24* functions as an oncogene promoting RCC growth. Indeed, an increase in TRIM24 expression was positively correlated with BMP8A and Nrf2 levels in human ccRCC specimens. It was shown that BMP8A contributed to increased Nrf2 phosphorylation, which stabilized ROS balance, thus promoting cell proliferation. Conversely, knockdown of *BMP8A* led to reduced Nrf2 activation and a consequent accumulation of ROS, inhibiting ccRCC cell proliferation and inducing apoptosis. Moreover, BMP8A aggravated ccRCC resistance to arsenic trioxide (As_2_O_3_) by modulating ROS levels and activating the Wnt signaling pathway. *BMP8A* knockdown downregulated TRIM24 expression and inhibited Wnt pathway activity, thus enhancing the chemosensitivity of ccRCC cells to As_2_O_3_ in both in vitro and in vivo models [123].

Several studies demonstrated that the KEAP1–Nrf2 signaling pathway is related to drug resistance in numerous tumors [124,125,126]. Indeed, a differential expression of Keap1 in tumor tissues compared to healthy adjacent tissues was reported: the *KEAP1* gene was inactivated or mutated in cancer tissues of patients with lung, liver, and ovarian cancer [127,128,129]. It was also evidenced that low expression of KEAP1 was correlated with a high mortality in patients with ovarian cancer [129]. Huang et al. investigated the role of KEAP1 in RCC and its effect on sensitivity to axitinib. In particular, axitinib inhibited RCC cell viability in a dose-dependent manner. The authors demonstrated that *KEAP1* silencing in ACHN cells led to a reduced sensitivity to axitinib, characterized by increased cell viability and decreased ROS production. The KEAP1–Nrf2 signaling pathway has been found to be implicated in drug resistance by diminishing the sensitivity of tumor cells to chemotherapeutics. In this study, axitinib treatment reduced Keap1 levels while stimulating Nrf2 expression and its downstream targets, NQO1 and HO-1. *KEAP1* silencing further enhanced Nrf2, NQO1, and HO-1 expression. Moreover, the study assessed ERK signaling, which has been linked to resistance against receptor tyrosine kinase inhibitors. It was observed a decreased phosphorylation of ERK in axitinib-treated cells, while Keap1 silenced cells did not show a change in ERK signaling, indicating that axitinib resistance induced by Keap1 silencing occurs independently of this pathway. To assess ACHN cell survival, gene silencing of *KEAP1* and *Nrf2*, as well as their overexpression, were performed. Knockdown of *KEAP1* increased viability in cells treated with axitinib, whereas *KEAP1* overexpression amplified the effects of the chemotherapeutic drug. Regulating KEAP1 could be a promising strategy to enhance therapeutic efficacy against drug resistance in RCC. Indeed, this study suggested that KEAP1 functions as a tumor suppressor in RCC, with decreased expression linked to poor prognosis and reduced sensitivity to axitinib [130].

Axitinib is emerging as a first-line treatment for metastatic RCC. The main obstacle is the development of resistance to the drug, which consequently compromises its effectiveness. The study of Huang et al. analyzed the role of the FXR1 in cellular resistance to axitinib in ccRCC. Axitinib-resistant and transfected cell lines were generated to study the interaction between FXR1 and the Keap1–Nrf2 pathway. Results showed that *FXR1* is overexpressed in axitinib-resistant cells and suppressed the stability of *KEAP1* mRNA, contributing to resistance. Reduction of FXR1 increased apoptosis and reduced drug resistance. In addition, knockdown of *KEAP1* inhibited autophagy and increased apoptosis, indicating that FXR1 promotes resistance by modulating the KEAP1–Nrf2 pathway; thus, FXR1 plays a key role in the resistance of ccRCC cells to axitinib [130]. The study of Ji et al. investigated the role of Nrf2/ARE pathway in influencing the biological characteristics of RCC cells and their sensitivity to targeted therapies. The authors demonstrated that expression levels of Nrf2, NQO1, and HO-1 are upregulated in RCC tissues and correlate with clinicopathological features. Transfection with shRNA targeting *Nrf2* in 786-O cells exhibited a decreased viability, reduced cell invasion and migration capability, and altered mRNA and protein levels of Nrf2, NQO1, HO-1, and glutathione transferase [131]. Moreover, they analyzed the response of cancer cells to treatment with sunitinib. In a previous study, Yang et al. demonstrated that sunitinib enhanced the apoptosis of medulloblastoma through the inhibition of the signal transducer and activator of transcription and PI3K-protein kinase B (AKT) signaling pathway [132,133]. Nrf2 regulates several genes involved in the PI3K-AKT pathway, which is essential for various biological processes, including cell proliferation, differentiation, and apoptosis. This pathway is also implicated in oncogenesis, cancer progression, and drug resistance [134]. Importantly, the PI3K-AKT pathway plays a critical role in resistance to sunitinib, making it a promising therapeutic target in renal cancer and other malignancies. This suggests that Nrf2 may enhance sunitinib resistance by activating the PI3K-AKT pathway, highlighting a potential mechanism through which tumor cells elude treatment. Indeed, after the transfection with shRNA targeting *Nrf2*, Ji et al. proved that cells showed increased sensitivity to sunitinib treatment. Future studies could further elucidate the potential of Nrf2 modulation in improving treatment outcomes for RCC patients [131]. Deng et al. investigated protein expression level of Nrf2/HO-1 in ccRCC. HO-1 is a protein that removes toxic heme and produces iron ions, biliverdin, and carbon monoxide. Disturbances in the proper levels of HO-1 are linked to various age-dependent disorders, including cancer [135]. Immunohistochemistry (IHC) was conducted to explore the expression patterns of Nrf2 and HO-1 in ccRCC and nearby normal tissues. The study aimed to evaluate their relationships with different clinicopathological factors and postoperative survival among ccRCC patients. Nrf2 and HO-1 expression levels were significantly elevated in ccRCC tissues compared to adjacent normal tissues. A notable correlation was found between Nrf2 protein levels and tumor size. Furthermore, higher expression levels of both Nrf2 and HO-1 were associated with poorer overall survival, suggesting their potential as prognostic indicators for ccRCC patients (Table 2) [136].

Angiogenesis is a process of fundamental importance in many physiological processes but is also involved in the progression of cancer [137,138]. For this reason, anti-angiogenic therapies may significantly improve the outcome in cancer patients. Pazopanib, a vascular endothelial growth factor receptor (VEGFR) inhibitor, is used for patients with advanced RCC who have not received prior systemic therapy. Wang et al. investigated the underlying mechanism through which pazopanib could promote cellular senescence in ACHN cell line. Cellular senescence is a mechanism that inhibits unlimited tumor proliferation, and it is characterized by accumulated changes in cell function and structure. In this study, they demonstrated that pazopanib induced cellular senescence, triggering the Nrf2/p53/plasminogen activator inhibitor (PAI) axis. In particular, in ACHN cells treated with pazopanib, an inhibition of viability and proliferation with an increased senescence-associated β-galactosidase (SA-β-Gal) activity and a reduced telomerase activity of telomerase were observed. Moreover, most cancers carry mutations in *p53*, an important cell cycle and apoptosis regulatory gene, thus causing an inactivation of *p53* function and preventing the entry of cancer cells into the process of cell senescence [139]. The authors have highlighted that p53/PAI signaling in ACHN cells was activated by pazopanib, implying that it can induce cell senescence in RCC cells through p53/PAI signaling activation. In addition, they observed that the Nrf2 level was particularly increased in pazopanib-stimulated ACHN cells. Therefore, *Nrf2* was knocked down in ACHN cells, and the results showed that silencing of *Nrf2* abrogated the ability of Pazopanib to promote cellular senescence and decrease telomerase activity [140].

c-Met is a receptor tyrosine kinase (RTK) that belongs to the MET family and is present on the surfaces of various cell types. The bond with its ligand, hepatocyte growth factor (HGF), triggers a cascade of intracellular signals involved in processes like embryogenesis and wound healing in healthy cells. However, in cancer cells, abnormal activation of the HGF/c-Met axis, often linked to *c-Met* gene mutations, overexpression, and amplification, drives tumor development and progression by stimulating multiple signaling pathways, including PI3K/AKT, Ras/MAPK, JAK/STAT, and Wnt/β-catenin [141]. *c-Met* is overexpressed and hyperactive in clear cell and papillary RCC, contributing to therapeutic resistance through the activation of the redox-sensitive transcription factor Nrf2 and cytoprotective HO-1. Although cabozantinib, a c-Met/RTK inhibitor, is approved for advanced RCC treatment, acquired resistance remains a significant challenge. The study of Rawat et al. investigated the potential of Honokiol, a natural phenolic compound, to downregulate c-Met-induced pathways. The combination of cabozantinib and Honokiol was found to synergistically inhibit renal cancer cell growth by increasing ROS production and promoting apoptosis and autophagy. Activation of c-Met was shown to induce Rubicon, a component of the class III PI-3K complex and a negative regulator of autophagy, as well as p62, which stabilizes Nrf2. Notably, the combination treatment significantly reduced levels of Rubicon, p62, and Nrf2 in RCC cells. The in vivo experiments in a tumor xenograft model showed that this combination inhibited renal tumor growth and was associated with reduced expression of Rubicon, p62, HO-1, and vessel density in tumor tissues. Overall, the cabozantinib and Honokiol combination effectively targets the c-Met-induced and Nrf2-mediated antioxidant pathway in renal cancer, enhancing oxidative stress and promoting tumor cell death [141]. A study of Zhao et al. focused on antitumoral properties of ginsenoside Rh4 (Rh4). The study aimed to explore the potential of Rh4 in increasing the sensitivity of RCC cells to ferroptosis and to clarify the mechanisms involved. Cell viability assays were conducted to assess how Rh4 influenced the sensitivity of 786-O and ACHN cell lines to RSL3-induced ferroptosis. Quantitative real-time polymerase chain reaction (qRT-PCR) was used to measure the expression levels of ferroptosis-related genes. Additionally, *Nrf2* was knocked down to investigate its role in mediating the effects of Rh4. They demonstrated that RAS-selective lethal 3 (RSL3) inhibited the progression of RCC cells by inducing ferroptosis. Additionally, Rh4 enhanced the sensitivity of RCC cells to ferroptosis triggered by RSL3. Rh4 downregulated the expression of ferroptosis-related genes, including superoxide dismutase 1, glutathione peroxidase 4, and catalase, effects that were diminished by *Nrf2* knockdown. These results indicated that Rh4 sensitized RCC cells to ferroptosis through the Nrf2 pathway by inhibiting Nrf2 signaling and reducing the expression of antioxidant enzymes. Thus, combining Rh4 with ferroptosis-inducing agents may offer a promising therapeutic strategy for treating RCC [142].

Disulfiram (DSF), used for the treatment of alcoholism, has potential as an antitumor drug [143]. Its antitumor activity is dependent on the presence of copper: diethyldithiocarbamate (DTC) is the primary metabolite of DSF and can form complexes with copper; these DSF/Cu complexes bind to and immobilize nuclear protein localization homolog 4 (NPL4), disrupting the P97-NPL4-UFD1 pathway, which is crucial for protein degradation, and ultimately resulting in cell death. Ni et al. demonstrated that DSF/Cu was highly toxic to RCC cells, since they inhibit their migration and invasion. The treatment disrupted mitochondrial homeostasis, leading to increased ROS and lipid peroxidation, which in turn caused oxidative stress and ferroptosis. While Nrf2 expression increased with DSF/Cu treatment, its mRNA levels remained unchanged, suggesting that Nrf2 degradation was slowed. At the same time, a decrease in NPL4 expression was observed. The overexpression of NPL4 was found to reverse Nrf2 levels and amplify DSF/Cu-induced oxidative stress and ferroptosis in RCC cells. Additionally, DSF/Cu demonstrated a synergistic effect with sorafenib in inhibiting RCC cell growth and ferroptosis in vivo. Overall, these findings suggested that targeting the compensatory increase in Nrf2 due to NPL4 inhibition enhances DSF/Cu-induced oxidative stress and ferroptosis in RCC (Table 2 and Table 3) [144].

**Table 2:** Drugs impacting Nrf2 activity.

Medicine	Brute Formula	Function	References
Axitinib	C_22_H_18_N_4_OS	Inhibits RCC cell viability in a dose dependent-manner. Selectively inhibits VEGF receptor tyrosine kinases 1, −2, and −3.	[130,145]
Cabozantinib	C_28_H_24_FN_3_O_5_	Inhibitor of c-Met/RTK. Approved for advanced RCC treatment.	[141]
Dimethyl fumarate	C_28_H_24_FN_3_O_5_	Disrupts the interaction between KEAP1 and Nrf2, allowing its nuclear translocation.	[90]
Disulfiram	C_10_H_20_N_2_S_4_	Suppresses cancer stem cells metabolism targeting aldehyde dehydrogenase. Inhibits proteasome activity forming complexes with metal ions. Induces apoptosis, inhibits cancer cell proliferation, and suppresses cancer cell metastasis.	[143]
Oltipraz	C_8_H_6_N_2_S_3_	Inhibits cancer formation. Allows nuclear Nrf2 translocation by disrupting its interaction with KEAP1.	[72,73]
Pazopanib	C_21_H_23_N_7_O_2_S	Inhibitor of vascular endothelial growth factor receptor (VEGFR). Used for patients with advanced RCC who have not received prior systemic therapy. Induces cellular senescence triggering Nrf2/p53/plasminogen activator inhibitor (PAI) axis.	[139]
Sunitinib	C_22_H_27_FN_4_O_2_	Performs the function of tyrosin kinese inhibitor on VEGFR 1, 2 and 3; PDGFR-α, and PDGFR-β.	[146]

**Table 3:** Nrf2 modulators.

Modulator	Mechanism	In Vitro/In Vivo	References
DMF	Nrf2 signaling activation can be adjusted by modulating DMF concentration.	In vitro	[147]
DJ-1	DJ-1 binds to Nrf2, preventing its association with KEAP1.	In vitro	[89]
SATB2	It regulates Nrf2 transcriptional activity on target genes.	In vitro	[120]
BMP8A	It enhances Nrf2 activation, increasing TRIM24 transcriptional activity.	In vivo and in vitro	[120]
NQO1 and HO-1	KEAP1 silencing enhances expression of Nrf2, NQO1 and HO-1.	In vitro	[130]
FXR1	A reduction of FXR1 increases apoptosis and reduces drug resistance. It promotes resistance by modulating the Keap-Nrf2 pathway.	In vitro	[130]
PI3K-AKT	Nrf2 enhances sunitinib resistance activating PI3K-Akt pathway.	In vitro	[133]

## 7. Conclusions

RCC is classified among the ten most common cancers worldwide, and its incidence is constantly increasing, making it an important burden for the healthcare systems. There is increasing evidence that Nrf2 activation exerts pro-tumorigenic effects, and alterations in Nrf2 signature in RCC are being studied. It is demonstrated that Nrf2 is linked not only to the progression of RCC but also to resistance to chemotherapy; thus, the Nrf2/KEAP1 pathway could be a promising target for the treatment of RCC. Given that chemotherapy is frequently ineffective or only marginally effective for RCC, research on compounds that can enhance chemotherapeutic drug effects is a primary goal.

Direct inhibition of Nrf2 activity in cancer cells has proven to be a significant challenge. Therefore, targeting upstream signaling pathways that regulate Nrf2 activation or focusing on downstream processes like metabolic reprogramming present more promising approaches, particularly when combined with other therapies. Additionally, with the rise of immunotherapies in cancer treatment, a deeper understanding of how Nrf2 influences immune functions in both cancer cells and stromal immune cells is crucial. This knowledge is essential for fully understanding Nrf2’s role in anti-tumor immune responses and for identifying potential targets to overcome resistance to immunotherapy [38]. In this review, we focused on the possible molecules which can be targeted to modulate the Nrf2 pathway, such as SATB2, BMP8A, FXR1, PI3K-AKT signaling, NQO1, HO-1, and c-Met.

Even if, to date, there are a few in vivo studies that demonstrate the effectiveness of targeting the Nrf2 pathway, the studies reported in this review suggest that not only could the modulation of Nrf2/KEAP1 pathway represent a valid strategy to counteract RCC progression but combining compounds with chemotherapeutic agents could also enhance the response to chemotherapy in clinical settings.

## Figures and Tables

**Table 1:** Main proteins impacting Nrf2 dysregulation in RCC.

Proteins	Role	References
KEAP1	Mutations affecting KEAP1 gene result in Nrf2 upregulation	[88]
PI3K	Activation of PI3K/AKT pathway induces Nrf2 activation	[32]
GSK-3β	Regulates Nrf2 degradation and nuclear export through phosphorylation mechanisms	[104]
AKT	Inhibits GSK-3β, resulting in Nrf2 nuclear accumulation	[105]
p62	Binds KEAP1 preventing the degradation of Nrf2	[106]
TGFβ1	Induces Nrf2 stabilization in a p-21 dependent manner	[99]
